# ProtRepeatsDB: a database of amino acid repeats in genomes

**DOI:** 10.1186/1471-2105-7-336

**Published:** 2006-07-07

**Authors:** Mridul K Kalita, Gowthaman Ramasamy, Sekhar Duraisamy, Virander S Chauhan, Dinesh Gupta

**Affiliations:** 1 Structural and Computational Biology Group, Malaria Group, International Centre for Genetic Engineering and Biotechnology (ICGEB), Aruna Asaf Ali Marg, New Delhi 110067, India; 2 Dana-farber Cancer Institute, Harvard Medical School, Dana-830, 44-Binney street, Boston, MA-02115, USA; 3 Malaria Group, International Centre for Genetic Engineering and Biotechnology (ICGEB), Aruna Asaf Ali Marg, New Delhi 110067, India

## Abstract

**Background:**

Genome wide and cross species comparisons of amino acid repeats is an intriguing problem in biology mainly due to the highly polymorphic nature and diverse functions of amino acid repeats. Innate protein repeats constitute vital functional and structural regions in proteins. Repeats are of great consequence in evolution of proteins, as evident from analysis of repeats in different organisms. In the post genomic era, availability of protein sequences encoded in different genomes provides a unique opportunity to perform large scale comparative studies of amino acid repeats. ProtRepeatsDB  is a relational database of perfect and mismatch repeats, access to which is designed as a resource and collection of tools for detection and cross species comparisons of different types of amino acid repeats.

**Description:**

ProtRepeatsDB (v1.2) consists of perfect as well as mismatch amino acid repeats in the protein sequences of 141 organisms, the genomes of which are now available. The web interface of ProtRepeatsDB consists of different tools to perform repeat s; based on protein IDs, organism name, repeat sequences, and keywords as in FASTA headers, size, frequency, gene ontology (GO) annotation IDs and regular expressions (REGEXP) describing repeats. These tools also allow formulation of a variety of simple, complex and logical queries to facilitate mining and large-scale cross-species comparisons of amino acid repeats. In addition to this, the database also contains sequence analysis tools to determine repeats in user input sequences.

**Conclusion:**

ProtRepeatsDB is a multi-organism database of different types of amino acid repeats present in proteins. It integrates useful tools to perform genome wide queries for rapid screening and identification of amino acid repeats and facilitates comparative and evolutionary studies of the repeats. The database is useful for identification of species or organism specific repeat markers, interspecies variations and polymorphism.

## Background

The function, type and size of amino acid repeat regions in proteins are vastly diverse. The amino acid repeats (hence forth referred as repeats and repeat containing proteins as repeat proteins) can be functionally neutral or active, tandem or scattered, and perfect or mismatch. Repeats may also range from a very minuscule to a highly significant fraction of proteins. Though functions of a few types of repeats are known, in general, function and evolution of a large variety of repeats are still poorly understood. Repeats have been broadly classified as perfect and mismatch repeats. The former class is further subdivided into homopeptide repeats (reiteration of any single amino acid, henceforth referred to as homo repeats) and heteropeptide repeats (repeats with different amino acids, henceforth referred to as hetero repeats). The mismatch repeats, consisting of repeats with substituted conserved amino acids, form the most diverse class of repeats.

Homo repeats containing proteins have been recognized as cause of several neurodegenerative and congenital malformation diseases. Proteins containing polyglutamine stretches (polyQ) have been observed in at least eight neural diseases [1]. Similarly, proteins with polyalanine tracts (polyA) are associated with various congenital malformations, skeletal dysplasia and nervous system anomalies [2]. Most of such homo repeats arise from trinucleotide slippage during replication [3,4], leading to proteins with homopolymeric tracts. Such proteins are either susceptible to misfolding, or aggregation and subsequent degradation, rendering them impaired.

Hetero repeats in proteins are quite diverse. For example, the glycine-proline amino acid run in eukaryotic genomes, polar zippers and prion like glutamine/asparagine-rich stretches [5-7], whereas mucins contain arrays of tandem repeats which are rich in serine and threonine residues corresponding to the most O-glycosylated part of the mature protein [8].

Functional studies have shown that the insertions of mismatch repetitive regions unique to a species can be indispensable to the functions of proteins, for example as shown in *Plasmodium berghei *glucose-6-phosphate dehydrogenase-6-phosphogluconolactonase, a bi-functional enzyme [9]. The role of repeats in antigenic variation and immune evasion by pathogens (e.g. *Plasmodium falciparum*, *Trypanosoma brucei *and group B *Streptococci*) has been elucidated [10-14]. Certain repeats provide structural scaffolds for structural packing of functional groups of amino acids to facilitate molecular interactions and stabilization. For example, Yeast Sup35p protein contains five oligopeptide repeats, which stabilizes its aggregation [15].

Identification of perfect repeats is simpler than that of mismatch repeats. All strategies to identify mismatch repeats have some limitation or the other, on account of polymorphism, low similarity and vast diversity of repeats [16]. Most strategies utilize algorithms based on Smith-Waterman local alignment, using substitution matrices [17,18], but some are also aimed to locate low complexity regions of proteins [19]. It is however clear that no single algorithm or strategy is sufficient to find all types of repeats in protein sequences.

Large-scale comparative studies of repeats across kingdoms will be required for better understanding of the role, diversity and evolution of repetitive sequences in proteins. Such comparisons may also provide deeper insight into the role of repeats in folding of proteins, immunogenicity and relevance to disease etiology [20]. However, most studies on repeats, so far, have focused either on a few classes of proteins in a limited number of organisms or only a particular type of repeat in different proteins or organisms [21,22]. Tools which can perform large scale cross species comparisons of different types of repeats are not yet developed. Here we present our efforts to build a robust protein repeats database and tools for large-scale analysis of repeats in different organisms. ProtRepeatsDB is a simple relational database to facilitate complex and large-scale comparative studies of repeats amongst organisms.

## Construction and contents

**Figure 1:**
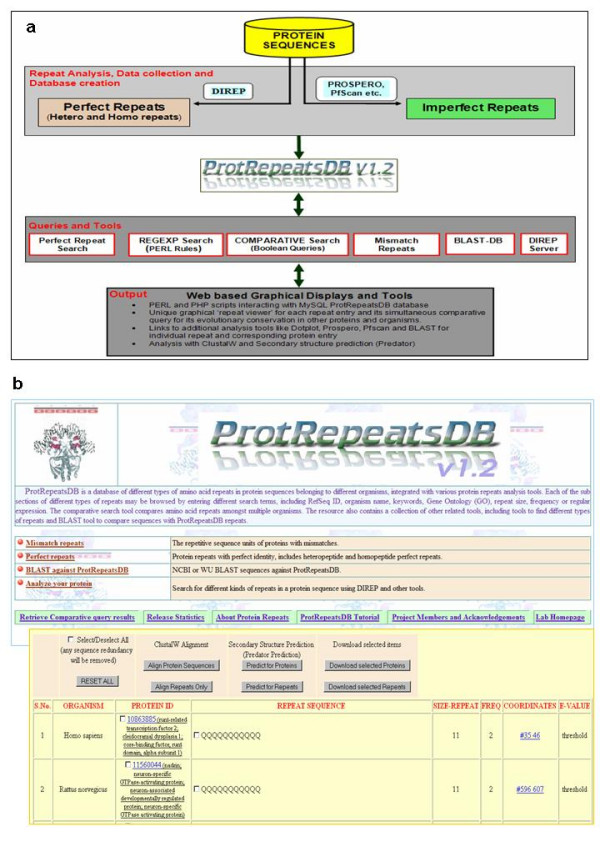
(a) Simplified schema of ProtRepeatsDB pipeline. (b) Snapshots of ProtRepeatsDB main page display, overlapped with the output obtained for analysis of the homo repeats in androgen receptors, see text for details.

ProtRepeatsDB is based on an underlying pipeline, shown in the Figure 1a. Repeats were identified using a script that automates sequence retrieval and execution of various repeat finders. All protein sequences used in the present study were obtained from the Reference Sequences (RefSeq) database [23] of NCBI (National Center for Biotechnology Information). It has been reported that for a protein of average size and composition, a run of an individual amino acid is statistically significant only if it is five or more residues long [24]. Hence, we used a cutoff size of five to identify repeats. A PERL program (DIREP) was used to detect tandem or scattered- hetero and homo repeats of size greater than or equal to five. Similarly, a different PERL code was used to detect homo repeats of size greater than five and less than ten. The mismatch repeats were identified using PROSPERO [25], which performs comparison of each sequence to itself, and prints all self alignments with p-values less than a predefined threshold. PROSITE patterns or profiles representing different amino acid repeats were identified using PFSCAN [26]. All the identified repeats and corresponding details were stored in a MySQL database. A large number of entries in the database were redundant, as several internal repeats which are part of larger repeats are detected as unique repeats. For example in the knob associated histidine rich protein (RefSeq:NP_472949) of *P. falciparum*, the repeats like KKKSK, KKKSKK, KKKSKKHKDN etc. were identified as separate repeats- whereas the longest repeat alone represents all the internal repeats. However, since such internal repeats may be conserved in other proteomes, this redundancy was removed by performing comparison of each repeat against the entire database for occurrence other than the self. The internal repeats were retained in the database only if present in at least one protein other than the self protein containing the repeat, otherwise only the longest stretch of the repeat, which represents all the internal repeats, was retained. This procedure significantly reduced the redundancy as well as the size of the database, without loosing any information regarding conservation of the repeats in different sequences and organisms.

## Utility and discussion

The ProtRepeatsDB and related tools facilitate retrieval and analysis of repeats in the database through different query pages and different sections. On submitting a query, a summary page of repeats satisfying the search conditions is generated. The summary page consists of a brief description of the sequences containing the repeat, such as the organism name, protein ID (hyperlinked to corresponding NCBI RefSeq entry), amino acids constituting the repeat, repeat size, repeat frequency, positions of the repeat in protein sequence (referred to as coordinates) hyperlinked to a detailed view, and E-value etc. (Figure 1b). Upon clicking coordinates of a repeat in the summary page, detailed information of the corresponding repeat is displayed as a new page consisting of two frames. The top frame consists of a dynamically generated color graph illustrating relative distribution of the repeat in the protein, followed by the sequence of the protein with repeats highlighted in red. In addition to this, links are provided to retrieve the sequence in FASTA format, perform repeat analysis using PROSPERO [25], DOTMATCHER [27], PfScan and BLAST [28] against ProtRepeatsDB. This section also provides links to display PROSITE matches, mismatch repeats and graphical representation of all the identified repeats in the protein sequence. The lower frame lists all the ProtRepeatsDB sequence entries sharing the same repeat.

**Figure 2:**
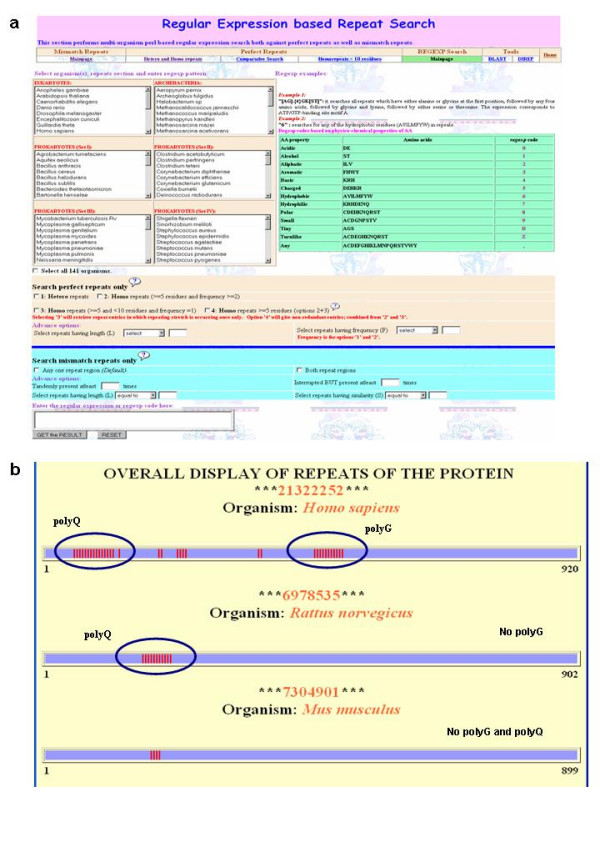
**ProtRepeatsDB**: (a) A snapshot of the REGEXP search tool: regular expression search can be performed on different ProtRepeatsDB sections. (b) Consolidated graphical view for all the proteins with conserved polyQ in androgen receptors (see text for more details).

The "REGEXP search" section accepts user-defined regular expressions to query the database. The regular expressions may either include patterns of single letter amino acid codes or patterns of predefined group codes based on similar physicochemical properties of amino acids. The details of predefined codes and groups are enumerated in the regular expressions query page (Figure 2a). For example, one can search the mismatch repeat section for prion repeat patterns 'P [HQ] [GS]G{1,3}WGQ', the octapeptide tandem repeat pattern associated with many neurological disorders like spongiform encephalopathies and dementia [29-31]. We used "REGEXP search" tool to query homo repeats section for glutamine and glycine homo repeats (also referred as polyQ and polyG respectively) (Figure 2a) repeats in rat, mouse and human proteomes. In the summary output page (not shown here), the NCBI RefSeq entry (RefSeq:21322252) corresponds to human androgen receptors (ARs). The human AR protein is characterized by presence of polyQ and polyG repeats, which are associated with gene polymorphisms, cryptorchidism [32] and a risk factor for prostate cancer [33]. However, we found that polyQ is absent in mouse AR and polyG is absent in both mouse (RefSeq:7304901) and rat (RefSeq:6978535) orthologous ARs (Figure 2b).

The comparative search section provides tools to compare repeats in multiple organisms using Boolean (AND or NOT) operators. For instance, by using 'AND' operator, one can find repeats common in different sets of organisms. Similarly, the "NOT" operator can be used to obtain a list of repeats which are mutually exclusive in user defined sets of organisms. Using the tools to search PROSITE repeats section, we investigated bacterial proteins with tetratricopeptide repeat (TPR), a structural repeat motif present in a wide range of proteins [34,35] and believed to be involved in protein-protein interactions and assembly of multi protein complexes [36,37]. We found that the TPR containing orthologs of *Vibrio vulnificus *CMCP6 [RefSeq:27367908], *Vibrio parahaemolyticus *[RefSeq:28901310] and *V. vulnificus *YJ016 [RefSeq:37676035] contain tandem glutamine repeats downstream from the TPR motifs. However, multiple alignments of the sequences revealed that the glutamine run is smaller in *Vibrio cholerae *[RefSeq:15600941] or interspersed, as in *Vibrio augustum *[RefSeq:90577283] and *Shewanella oneidensis *MR-1 [RefSeq:24374614]. Significance of the glutamine homo repeats in *Vibrio *proteins is not clear; however such glutamine repeats are known to induce formation of quasi-aggregates in the early stage of amyloid protein fibrillization [38] and inhibition of protein degradation [39] in various proteins associated with human neurodegenerative disease.

Overall analysis of homo repeat proteins in different proteomes was performed by dividing them into two groups of varying repeat sizes and frequency of occurrence. First group consists of proteins with smaller homo repeats of size less than ten, occurring one or more times in a protein sequence. Second group consists of proteins with longer homo repeats of size ten and greater, occurring more than once in a protein sequence. Table 1 summarizes the comparative distribution of homo repeat proteins in different proteomes in the two groups mentioned above. The table lists first five and first three proteomes, if ordered according to decreasing percentages of proteins in the first and second group, respectively. The second group list was restricted to first three organisms only, mainly because of the fact that there are not many proteins which contain extremely large as well as repeating homo repeats. For homo repeats of each of the amino acids, the corresponding number of protein sequences (brackets) as percentage of all the homo repeats in the organism is listed (square brackets). Table 1 brings out certain interesting facts, for example amino acid preference for homo repeats in different organisms. The asparagine homo repeats are most abundant in *P. falciparum *proteins. In *P. falciparum *proteome, we found that 1675 proteins contain asparagine homo repeats of size less than ten, and as many as 280 proteins *P. falciparum *proteins have asparagine repeats of size ten and above, occurring more than once. The proteins with asparagine repeats constitute around 31 percent of the total annotated proteins in the parasite. The abundance of asparagines repeats has been correlated with many prion-like domains in the parasite genome [40]. We also found that the proteins with lysine homo repeats also constitute a large percentage (47 percent) of homo repeat containing proteins in *P. falciparum*, which is 21 percent of all the annotated proteins in the genome. However, there are only two *P. falciparum *proteins with lysine homo repeats in the second group. The ProtRepeatsDB comparative tools also reveal that the number of glutamic acid homo repeat proteins is higher than those containing aspartic acid in all the eukaryotes, except *P. falciparum*.

Glutamine homo repeat proteins are most abundant in *D. melanogaster*, accounted by 1582 proteins, which is 34 percent of all the homo repeat proteins in the genome. The polar glutamine repeats are primarily present in proteins that are involved in transcription-translational activities especially proteins interacting with DNA and other proteins. Proline homo repeats of size ten and greater are present in thirteen proteins of *H. sapiens*, ten *A. thaliana *proteins, and four *M. musculus *proteins. One class of such proteins includes formin-like proteins, common to the above three organisms, which are involved in processes such as morphogenesis, embryonic differentiation, cell polarity, and cytokinesis [41]. The proline-rich regions of formin-like proteins are believed to be involved in protein-protein interactions as exemplified by the crystal structure of the binding domain in formin binding protein (FBP11) with specific binding to PPLPp motifs in the formins [42].

The use of ProtRepeatsDB in comparative analysis of homo repeat proteins in related species is perhaps best illustrated by comparison of such repeats in *H. sapiens, R. norvegicus *and *M. musculus*. The most abundant homo repeat proteins in human, mouse and rat have repeats of glutamic acid, proline, leucine, alanine, glutamine, glycine, serine or lysine residues. Homo repeat proteins containing repeats of isoleucine, asparagine and valine are miniscule in *H. sapiens *and related species. Number of phenylalanine homo repeat proteins in *M. musculus *is relatively higher than those in rat and human proteomes. The percentage of cysteine, methionine and arginine homo repeat protein is almost same in all the three organisms. There is no tyrosine homo repeat protein in rat and mouse; however, there is one tyrosine homo repeat in a *H. sapiens *helicase protein. The mixed-lineage leukemia proteins of human, which are also trithorax homologs of *Drosophila*, have homo repeats of serine, glutamic acid, proline and glycine. A human and mouse ortholog of the protein has a serine run of forty two and thirty amino acid residues respectively, however the run is absent in the corresponding rat ortholog. The non trithorax homologs in human mixed-lineage leukemia proteins also lack serine runs.

The Table 1 also reveals that there is no histidine, cysteine, tyrosine, isoleucine and tryptophan homo repeat falling under second group, encoded in any of the genomes. The rare methionine homo repeats have been observed in proteins belonging to *A. thaliana*, *A. gambiae *and *P. falciparum*. In fact, a CHP-rich zinc finger protein (CHP-rich: cysteine, histidine and proline rich) of *A. thaliana *contains methionine repeat of size 11, which also happens to be the longest homo repeat of methionine in the database. Phenylalanine and tyrosine repeats are present in several *P. falciparum *hypothetical proteins and putative proteins like succinyl-CoA synthetase alpha subunit, syntaxin, Ser/Thr protein kinase, sequestrin and acid phosphatase. Glycine homo repeat proteins are ubiquitously present in almost all super kingdoms. The mycobacterial parasite *M. bovis *has the highest number of alanine homo repeat proteins, representing 64.5 percent of its homo repeat proteins and nearly 4.8 percent of annotated proteins in the genome.

**Figure 3:**
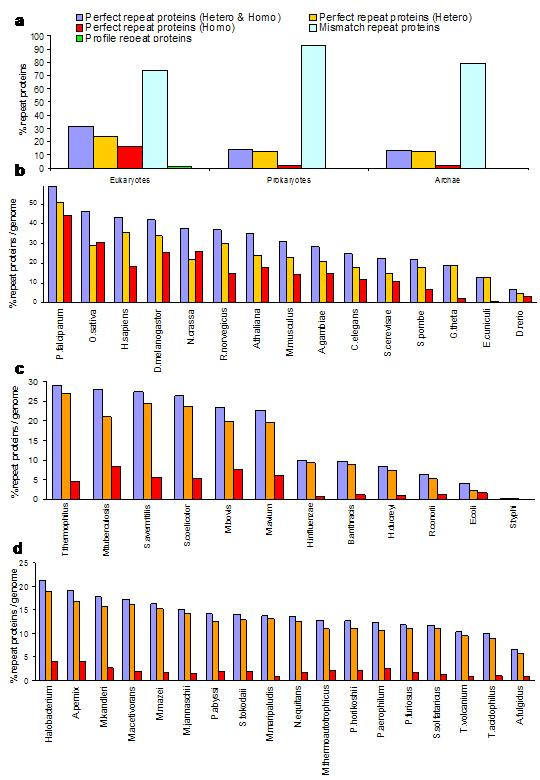
**Comparative distribution of different types of repeat proteins in ProtRepeatsDB**. (a) The percentage of repeat proteins in three super kingdoms containing perfect repeats (including hetero and homo repeats), perfect hetero repeats, perfect homo repeats, mismatch repeats and repeats with PROSITE repeat profiles. (b) The percentage of different types of perfect repeat proteins in few eukaryotic genomes in ProtRepeatsDB. (c) The percentage of different types of perfect repeat proteins in few prokaryotic genomes. (d) The percentage of different types of perfect repeat proteins in few archeal genomes. **Note**: figures b-d: The organisms with 6 highest and 6 lowest repeat protein percentages are shown here.

Statistical analysis of ProtRepeatsDB is in agreement with the earlier observation that the percentage of perfect repeat containing repeat proteins is higher in eukaryotes than in prokaryotes and archeals (Figure 3a). Our analysis indicates that hetero repeat proteins also follow a similar trend, although, homo repeats are a predominant feature of the eukaryotic genomes (Figure 3a). Similarly, the PROSITE profiles representing repeats are mainly found in eukaryotes. The high percentage of mismatch repeats amongst all super kingdoms indicates duplication events during evolution.

Figure 3b–d, is a graphical representation of perfect repeats in representative proteomes. Figure 3b gives the distribution of repeat proteins in the eukaryotes, which indicates that *P. falciparum *has an unusual distribution of repeats in comparison with other eukaryotes; it is the only organism with more than 50% of its proteome constituted by proteins containing different kinds of repeats. Since one of the important role of repeat proteins is believed to be in immune evasion by the parasites, we investigated the nature of repeats in immuno dominant proteins in *P. falciparum*, and we found that the parasite antigens are not only rich in homo repeats, several of these proteins are also marked by abundance of hetero repeats. We also found that twenty one antigens of the parasite also have hetero repeats of size ten or greater. Noticeably, most of these hetero repeat regions are rich in charged or polar amino acids, which are known to play important role in antigen-antibody or antigen-carrier protein interactions. There are several such examples, including that of the S-antigen in *P. falciparum*, which is characterized by the presence of two similar repeats of sixty nine residues each, forming the coiled and surface exposed regions of the proteins. Similarly, other plasmodium antigens such as the LSA (liver stage antigen), antigen 332, MESA (mature-parasite- infected erythrocyte surface antigen) or PfEMP2 (*P. falciparum *erythrocyte membrane protein 2) and FIRA (interspersed repeat antigen) also have large inserts of hetero repeats. MESA and ring-infected erythrocyte antigen are surface exposed antigens rich in glutamic acid and lysine residues. Some of these antigens are also being developed as vaccines [43].

Amongst other eukaryotes, we found abundant repeat proteins in *O. sativa*, *H. sapiens*, *D. melanogastor *and *Neurospora crassa*. Interestingly, *N. crassa *and *O. Sativa *are the only organisms in the database, in which the percentage of homo repeat proteins exceeds that of hetero perfect repeat proteins. In *Guillardia theta *and *Encephalitozoon cuniculi*, the percentage of all types of perfect repeat proteins is almost the same as that of perfect hetero repeat proteins alone, which implies that the number of homo repeat proteins is quite less and in fact lowest amongst the eukaryotes studied here. However, it is warned that several RefSeq genomes are still under review and contain several proteins sequences yet to be verified, and *G. theta *and *E. cuniculi *genomes are examples of such genomes.

Rat and mouse proteins have lower number of perfect repeat proteins as compared to human proteome (Figure 3b). However, percentage of homo repeat proteins in rat and mouse are almost same and only marginally lower than that of humans, implying that the human proteome is richer in proteins with hetero repeats. It might have some implications regarding the observation that the generation of repeats promote protein evolution [44] and formation of novel functional variants [45,46]. We observed differential distribution of repeats in other closely related species as well, for example *Saccharomyces cerevisiae *and *S. pombe *have almost equal fraction of repeat proteins, however, the percentage of homo repeat proteins is considerably higher in *S. cerevisiae *(11%) as compared to *S. pombe *(~7%)(Figure 3b).

The percentage of repeat proteins in prokaryotic genomes is lower as compared to that in eukaryotes. The highest percentage of repeat proteins in prokaryotes is observed in *Thermus thermophilus *(~29%) followed by *Mycobacterium tuberculosis Rv *(~28%) whereas *Salmonella typhi *contains the lowest number of repeat proteins not only amongst prokaryotes, but in all the proteomes studied here (Figure 3c). It is interesting to note that *M. tuberculosis Rv *has higher percentage of repeat proteins as compared to that in other mycobacterium species, namely *M. bovis *and *Mycobacterium avium*. All the mycobacterial species except *Mycobacterium leprae*, predominantly have alanine, glycine and proline homo repeat proteins. Alanine repeats are abundant in PPE (proteins with proline-proline-glutamine motifs) family and a few PE-PGRS proteins (proteins with proline-glutamine motifs and polymorphic GC-rich repetitive sequences) [47] of *M. tuberculosis Rv *and *M. bovis*. However, all *M. avium *alanine homo repeat proteins are hypothetical proteins except two: one PE family protein and a PstA (phosphate-specific transport subunit A) protein. *M. leprae *has the lowest percentage of repeat proteins amongst mycobacterium genomes in the database mainly due to lower numbers of PPE family homologs. The glycine repeats are exclusively present in PE-PGRS proteins of *M. tuberculosis Rv *and *M. bovis *but absent in *M. avium *and *M. leprae*. The PE-PGRS is a newly identified family of fibronectin-binding proteins involved in antigenic variation. The number of glycine repeat proteins in *M. avium *and *M. leprae *is much less as compared to that in the other two species. All the four species of mycobacterium have proline repeats, mainly in the proline-rich antigens. Repetitive regions have been used as genetic markers for the strain differentiation and epidemiology of mycobacterium species [48,49]. It will be interesting to perform systematic comparative investigation of different types of repeats in the species and investigate possible roles of repeats in specific interactions with their hosts.

Amongst the archeal genomes, the percentage of total repeat proteins is highest in *Halobacterium *(~21%) followed by *Aeropyrum pernix *and the lowest in *Archaeoglobus fulgidus *(~7%) (Figure 3d). Notably, we found that all archeal proteomes have lower number of homo repeat proteins. The highest percentage of homo repeat proteins is observed in *Halobacterium *sps. (~4%) and lowest in *A. fulgidus *(~1%). During evolution, archeal genomes mainly accumulated hetero repeat proteins but the resistance towards evolution of homo repeat proteins or mechanism of specific selection of hetero duplication in genomes is not yet known. A detailed and comprehensive analysis of different types of repeats in the three super kingdoms as well as intra-kingdom might provide leads to answers of many open questions, which are, however, beyond the scope of the present study.

The ProtRepeatsDB contains tools for large-scale comparison of protein repeats across genomes to aid studies related to evolution of repeat genesis and functional roles of such repeats in different organisms. The differential propensity of repeats among the super kingdoms/lineages and organisms within the same kingdom/genus emphasizes that different types of repeats have undergone different selection pressure and propagation mechanisms during evolution. However, conclusions derived from analysis of sequences emerging from the sequencing projects with little or no manual curation or additional experimental validation of gene structure have to be dealt with additional care, as some of such sequences may have translations from incorrect gene predictions.

## Conclusion

ProtRepeatsDB is a multi-organism database of protein repeats, which is the first database of its kind that incorporates different kinds of repeats viz. perfect repeats-homopeptides and heteropeptides, mismatch repeats and profile patterns representing different families of repeats. The current version (v 1.2) consists of 120686 perfect repeats, 834621 mismatch repeats and 3673 profile repeats from 894890 protein sequences belonging to 141 genomes. The web interface of ProtRepeatsDB consists of unique tools which allow formulation of queries for retrieval and cross species comparison of repeats.

## Availability and requirements

ProtRepeatsDB is freely accessible on the Internet at  (Figure 1b). The database is divided into integrated sections of different types of repeats for easy browsing and data retrieval. The web interface of ProtRepeatsDB is supported with PERL and PHP scripts which enable formulation of queries against the database. Results are displayed either in tabulated or graphical formats.

## Authors' contributions

Corresponding author- DG, and VSC conceived the project. DG wrote the manuscript, MKK and VSC reviewed it. MKK, SD, GR and DG developed the MySQL database, Web interface and related PHP scripts. GR and DG developed DIREP.

## Future directions

ProtRepeatsDB will be regularly updated with protein repeat sequences in emerging annotated sequences from various genome sequencing projects. ProtRepeatsDB will be developed further to include cross links with other databases, repeats detected by other repeat finding algorithms, 3-dimensional structures of repeat proteins, web based repeat finding servers, tools for phylogenetic analysis and ortholog based search for comparative analysis of repeats.

## Figures and Tables

**Table 1:** Relative distribution of homo repeats (tandem or scattered) of size <10 (occurring one or more times) and ≥10 (occurring more than once). The figures within round brackets represent the number of homo repeat proteins of corresponding amino acid and organism. The figures within square brackets represent percentage of homo repeat proteins of corresponding amino acid and organism, to that of all the homo repeat proteins in the organism.

	**Amino Acid**	**Proteins with homo repeats of size <10**					**Proteins with homo repeats of size ≥10**		
			
		**1^st^**	**2^nd^**	**3^rd^**	**4^th^**	**5^th^**	**1^st^**	**2^nd^**	**3^rd^**
	
**Acidic (Polar)**	**D**	Sce(94) [13.33]	Pfa(270) [11.56]	Ncr(280) [9.18]	Dre(81) [8.7]	Ara(395) [7.7]	Sce(3) [0.43]	Pfa(6) [0.44]	Ara(3) [0.39]
	**E**	Hsa(996) [21.95]	Mus(761) [20.35]	Dre(187) [20.08]	Rat(654) [19.53]	Spo(53) [15.54]	Mus(28) [4.11]	Hsa(25) [2.43]	Rat(9) [1.48]
**Basic (Polar)**	**R**	Osa(545) [12.5]	Sav(48) [11.11]	Sco(44) [10.06]	Rat(161) [4.81]	Mus(151) [4.03]	Mus(1) [0.15]	--	--
	**K**	Pfa(1100) [47.11]	Lin(12) [19.6]	Spo(37) [10.8]	Dre(92) [9.88]	Sce(59) [8.37]	Ara(1) [1.43]	Ncr(3) [0.56]	Pfa(2) [0.14]
	**H**	Ano(153) [6.28]	Dme(282) [6.06]	Ncr(75) [2.87]	Cel(68) [2.69]	Ara(135) [2.63]	--	--	--
**Polar**	**S**	Lpl(25) [36.23]	Ara(1742) [33.99]	Spo(112) [32.84]	Sce(194) [27.52]	Dre(221) 23.73]	Hsa(20) [1.94]	Mus(9) [1.32]	Dme(14) [0.76]
	**T**	Lpl(14) [20.28]	Cel(307) [12.15]	Ncr(316) [12.09]	Dme(516) [11.11]	Ano(235) [9.65]	Dme(6) [0.33]	Ano(2) [0.35]	Rat(1) [0.16]
	**N**	Pfa(1675) [71.73]	Sce(88) [12.48]	Dme(465) [9.99]	Ncr(146) [5.58]	Ara(234) [4.56]	Pfa(280) [20.29]	Ncr(8) [1.49]	Dme(2) [0.11]
	**Q**	Dme(1582) [34.01]	Lma(25) [31.6]	Ano(576) [23.65]	Ncr(525) [20.09]	Sce(129) [18.30]	Dme(131) [7.12]	Ncr(34) [6.34]	Hsa(43) [4.18]
	**C**	Mus(25) [0.66]	Dre(6) [0.64]	Rat(21) [0.62]	Ano(11) [0.45]	Hsa(21) [0.44]	--	--	--
	**Y**	Pfa(30) [1.28]	Ype(1) [1.23]	Cel(7) [0.27]	Ano(3) [0.12]	Osa(5) [0.11]	--	--	--
**Non-polar**	**G**	Osa(1294) [26.69]	Ano(601) [24.68]	Dme(957) [20.57]	Ncr(454) [17.38]	Mbo(47) [15.87]	Ara(11) [1.43]	Has(10) [0.97]	Dme(8) [0.44]
	**A**	Mbo(191) [64.52]	Sav(222) [51.38]	Sco(223) [51.02]	Osa(1520) [34.87]	Dme(1209) [25.98]	Dme(34) [1.85]	Mus(10) [1.47]	Hsa(11) [1.07]
	**V**	Osa(79) [1.89]	Ano(29) [1.19]	Rat(35) [1.04]	Ara(53) [1.03]	Cel(23) [0.91]	Rat(1) [0.10]	--	--
	**L**	Tth(48) [49.48]	Dra(49) [42.61]	Pae(49) [32.23]	Rat(662) [19.77]	Hsa(860) [18.26]	Xfa(1) [9.09]	Ano(1) [0.18]	--
	**I**	Sto(7) [12.72]	Pfa(18) [0.77]	Dre(7) [0.75]	Mus(26) [0.69]	Cel(14) [0.55]	--	--	--
	**P**	Hsa(851) [18.07]	Osa(712) [16.33]	Mus(608) [16.21]	Cel(394) [15.59]	Rat(506) [15.11]	Ara(10) [1.30]	Has(13) [1.26]	Mus(4) [0.59]
	**M**	Ara(16) [0.33]	Dre(3) [0.32]	Pfa(5) [0.21]	Ano(5) [0.20]	Ncr(3) [0.11]	Ara(1) [0.13]	--	--
	**F**	Pfa(65) [2.78]	Ara(43) [0.83]	Cel(19) [0.75]	Mus(23) [0.61]	Osa(16) [0.37]	Mus(1) [0.15]	Osa(1) [0.13]	--
	**W**	Ano(3) [0.12]	Osa(3) [0.06]	Ncr(1) [0.03]	--	--	--	--	--

**Note**: **Ano**: *Anophelis gambiae*; **Ara**: *Arabidopsis thaliana*; **Cel**: *Caenorhabditis elegans*; **Dre**: *Danio rerio*; **Dra**: *Deinococcus radiodurans*; **Dme**: *Drosophila melanogastor*; **Hsa**: *Homo sapiens*; **Lpl**: *Lactobacillus plantarum*; **Lma**: *Leishmania major*; **Lin**: *Leptospira interrogans*; **Mus**: *Mus musculus*; **Mbo**: *Mycobacterium bovis*; **Ncr**: *Neurospora crassa*; **Osa**: *Oryza sativa*; **Pfa**: *Plasmodium falciparum*; **Pae**: *Pseudomonas aeruginosa*, **Rat**: *Rattus norvegicus*; **Sce**: *Saccharomyces cerevisiae*; **Spo**: *Schizosaccharomyces pombe*; **Sav**: *Streptomyces avermitilis*; **Sco**: *Streptomyces coelicolor*; **Sto**: *Sulfolobus tokodaii*; **Tth**: *Thermus thermophilus*; **Ype**: *Yersinia pestis*; **Xfa**: *Xylella fastidiosa*
